# Impacts of Aortic Occlusion Duration ≥90 Minutes on Short- and Long-Term Outcomes in Infectious Endocarditis

**DOI:** 10.31083/RCM47654

**Published:** 2026-07-20

**Authors:** Jing-bin Huang, Ang Tang, Chang-chao Lu, Zhao-ke Wen

**Affiliations:** ^1^Department of Cardiothoracic Surgery, The People’s Hospital of Guangxi Zhuang Autonomous Region, Guangxi Academy of Medical Sciences, 530000 Nanning, Guangxi, China; ^2^Department of Ultrasound, Ruikang Hospital Affiliated to Guangxi University of Traditional Chinese Medicine, 530000 Nanning, Guangxi, China

**Keywords:** infectious endocarditis, aortic occlusion length ≥90 minutes, risk factor, long-term survival

## Abstract

**Background:**

To determine the impacts of aortic occlusion duration ≥90 minutes on short- and long-term survival in patients with infectious endocarditis (IE).

**Methods:**

We retrospectively reviewed patients who underwent surgical treatment for IE at our hospital.

**Results:**

In the present study, 368 patients had an aortic occlusion duration ≥90 minutes (45.2%). Aortic occlusion length ≥90 minutes was associated with in-hospital mortality. A duration from symptom onset to operation ≥1 month, vegetation diameter ≥10 mm, cardiopulmonary bypass (CPB) time ≥180 minutes, preoperative left ventricular end-diastolic diameter (LVEDD) ≥52 mm, preoperative aortic regurgitation ≥4 cm^2^, and preoperative mitral regurgitation (MR) ≥8 cm^2^ were associated with an aortic occlusion duration ≥90 minutes. Aortic occlusion duration ≥90 minutes was also significantly associated with 1- and 5-year mortality after cardiac operation and was also a significant risk factor for long-term survival. Cox proportional hazards regression for all-cause mortality identified symptom-to-operation interval ≥1 month, vegetation size ≥10 mm, aortic occlusion duration ≥90 minutes, and red blood cell transfusion volume ≥2 units as associated with all-cause mortality. There was a significant positive correlation between CPB duration and aortic occlusion duration. A CPB duration >120.5 minutes had 74.5% sensitivity and 61.9% specificity for diagnosing an aortic occlusion duration ≥90 minutes.

**Conclusions:**

In our investigation, an aortic occlusion duration ≥90 minutes in patients with infectious endocarditis was a significant risk factor for both short- and long-term mortality.

## 1. Introduction

Infectious endocarditis (IE) is a kind of infection of the cardiac valve and inner layer of the cardiac cavity. It is still a dangerous disease. The mortality rate within one year is close to 30%, and the global incidence rate of IE is still rising [1,2,3,4]. It has been shown that aortic occlusion length and cardiopulmonary bypass (CPB) duration during cardiac surgery are associated with postoperative mortality and the incidence rate of complications. The increase in aortic occlusion length is associated with postoperative low cardiac output, prolonged mechanical ventilation, acute kidney injury (AKI), and neurological deficits. Because of the various adverse effects resulting from prolonged aortic occlusion, the prognosis of mid-term survival rate following complicated surgery is poor [5,6,7,8]. “Aortic occlusion length” is synonymous with “aortic cross-clamp time” in our paper here, which is the standard term in the field in our study.

Investigations of aortic occlusion length ≥90 minutes on long-term survival in IE are rare. The objective of this research was to investigate the impact of aortic occlusion length ≥90 minutes on short- and long-term survival in IE.

## 2. Materials and Methods

### 2.1 Data Source 

This study includes data of IE patients between 2006 and 2022 collected from the database of our hospital. Diagnosis was based on modified Duke criteria. The Ethical Committee of our institution approved the research.

### 2.2 Variables 

Parameters were investigated (data in **Supplementary Material**).

### 2.3 Follow-Up

Echocardiogram, electrocardiogram, and X-ray chest film were investigated for all patients from discharge to date of death or the end date of the research once every 3 to 12 months. The patients were interviewed at the outpatient department or contacted by phone or WeChat at the last time follow-up.

### 2.4 Statistical Analyses

We reported categorical data as percentages and quantitative variables as the mean ± standard deviation (SD). We also adopted the χ^2^ test, Fisher’s Exact Test, and the unpaired *t*-test. Logistic regression analyses were performed. Kaplan–Meier curves were completed and compared by the log-rank test. A Cox proportional hazards analysis was performed. All tests were 2-sided, and significance was assessed at *p* < 0.05. Data were investigated by IBM SPSS Statistics 24.0 (IBM City, Armonk, NY, USA).

## 3. Results

**Fig. 1.**
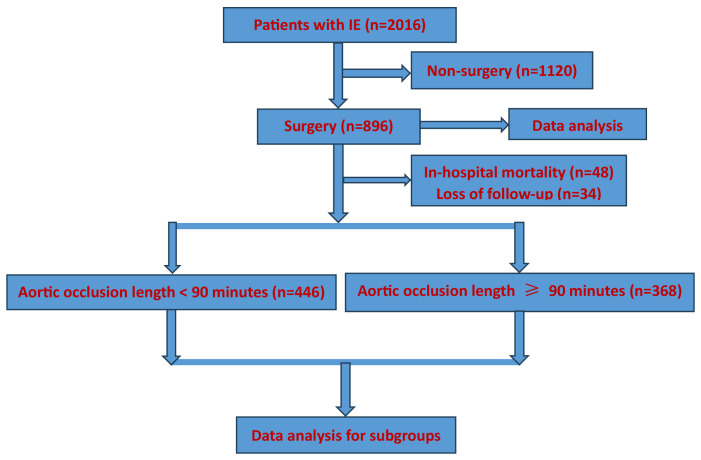
**Flow chart of patients in the study**. IE, infectious endocarditis.

896 patients with IE who underwent surgical treatment were included. 814 patients were included in the subgroup for further analysis, excluding 48 cases that died in hospital and 34 cases that were lost to follow-up from the subgroups. The aortic occlusion length of the in-hospital death group (n = 48) and the survival group (n = 848) were 115.67 ± 5.96 and 89.75 ± 1.18 minutes, respectively (*p* < 0.001). The capacity of aortic occlusion length for predicting all-cause mortality in follow-up was evaluated by calculating the receiver operating characteristic (ROC) curve. Using 90 minutes as a cutoff level, a value of aortic occlusion length ≥90 minutes was 66.7% sensitive and 63.6% specific for the diagnosis of all-cause mortality in follow-up, with an ROC curve 0.700 (95% confidence interval: 0.654–0.745; *p* < 0.001) and a Youden index 0.317. We also incorporated previous literature utilizing 90 minutes as a threshold for risk in complex cardiac surgery [9,10,11,12,13]. We decided to take 90 minutes as the cut-off value for grouping and comparing the differences between the groups with aortic occlusion length < and ≥90 minutes (Fig. 1).

### 3.1 Preoperative

Compared with the group with aortic occlusion length <90 minutes, male gender (80.0% vs 54.7%, *p* < 0.001), age (41.58 ± 14.61 vs 35.91 ± 13.51 years, *p* < 0.001), duration between symptoms and operation (2.74 ± 2.51 vs 2.23 ± 1.91 months, *p* = 0.001), vegetation size (11.22 ± 6.50 vs 9.36 ± 6.71 mm, *p* < 0.001), preoperative left ventricular end-diastolic diameter (LVEDD) (62.18 ± 8.02 vs 60.14 ± 10.13 mm, *p* = 0.001), preoperative aortic regurgitation (7.23 ± 6.64 vs 4.11 ± 6.61 cm^2^, *p* < 0.001), preoperative mitral regurgitation (MR) (7.75 ± 5.22 vs 6.88 ± 6.90 cm^2^, *p* = 0.037) preoperative tricuspid regurgitation (5.08 ± 4.25 vs 4.34 ± 5.06 cm^2^, *p* = 0.020), and serum creatinine before surgery (85.07 ± 30.93 vs 78.03 ± 35.38 µmol/L, *p* = 0.002) were significantly increased in the aortic occlusion length ≥90 minutes group (Table 1).

**Table 1.** **Comparison of groups with aortic occlusion length < and ≥90 minutes (n = 896)**.

Variable		Group of aortic occlusion length <90 minutes (n = 495)	Group of aortic occlusion length ≥90 minutes (n = 401)	*p* value
Preoperative				
	Male gender, n (%)	271 (54.7%)	321 (80.0%)	<0.001
	Age, years	35.91 ± 13.51	41.58 ± 14.61	<0.001
	Weight, kg	56.23 ± 12.53	54.55 ± 10.93	0.035
	Duration between symptoms and operation, months	2.23 ± 1.91	2.74 ± 2.51	0.001
	Vegetation size, mm	9.36 ± 6.71	11.22 ± 6.50	<0.001
	Preoperative LVEDD, mm	60.14 ± 10.13	62.18 ± 8.02	0.001
	Preoperative LVEF, %	62.28 ± 7.92	61.55 ± 7.47	0.166
	Preoperative aortic regurgitation, cm^2^	4.11 ± 6.61	7.23 ± 6.64	<0.001
	Preoperative MR, cm^2^	6.88 ± 6.90	7.75 ± 5.22	0.037
	Preoperative tricuspid regurgitation, cm^2^	4.34 ± 5.06	5.08 ± 4.25	0.020
	Serum creatinine before surgery, µmol/L	78.03 ± 35.38	85.07 ± 30.93	0.002
Operative				
	In-hospital mortality, n (%)	15 (3.0%)	33 (8.2%)	0.001
	CPB time, minutes	102.83 ± 23.62	187.08 ± 37.30	<0.001
	Mechanical ventilation time, hours	41.09 ± 61.13	56.86 ± 58.03	<0.001
	Length of ICU stay, days	4.17 ± 2.95	5.46 ± 2.83	<0.001
	Postoperative hospital stay, days	17.46 ± 5.98	20.78 ± 9.20	<0.001
	Creatinine in serum 24 h after surgery, µmol/L	84.01 ± 39.84	93.88 ± 46.63	0.001
	Creatinine in serum 48 h after surgery, µmol/L	90.59 ± 61.84	120.92 ± 73.85	<0.001
	AKI, n (%)	79 (16.0%)	193 (48.1%)	<0.001
	Chest drainage, mL	628.20 ± 458.89	621.05 ± 309.85	0.790
	Postoperative LVEDD, mm	47.25 ± 7.62	48.78 ± 6.19	0.001
	Postoperative LVEF, %	58.97 ± 7.64	58.87 ± 5.78	0.824
	Frozen plasma transfusion, mL	626.59 ± 523.72	615.06 ± 386.95	0.714
	Red blood cells transfusion, units	2.45 ± 3.58	3.04 ± 2.63	0.006

LVEDD, left ventricular end-diastolic diameter; CPB, cardiopulmonary bypass; ICU, intensive care unit; LVEF, left ventricular ejection fraction; AKI, acute kidney injury; MR, mitral regurgitation.

### 3.2 Operative

Compared with the group with aortic occlusion length <90 minutes, in-hospital mortality (8.2% vs 3.0%, *p* = 0.001), extracorporeal circulation time (187.08 ± 37.30 vs 102.83 ± 23.62 minutes, *p* < 0.001), mechanical ventilation time (56.86 ± 58.03 vs 41.09 ± 61.13 hours, *p* < 0.001), length of intensive care unit (ICU) stay (5.46 ± 2.83 vs 4.17 ± 2.95 days, *p* < 0.001), postoperative hospital stay (20.78 ± 9.20 vs 17.46 ± 5.98 days, *p* < 0.001), creatinine of serum 24 h after surgery (93.88 ± 46.63 vs 84.01 ± 39.84 µmol/L, *p* = 0.001), creatinine of serum 48 h after surgery (120.92 ± 73.85 vs 90.59 ± 61.84 µmol/L, *p* < 0.001), postoperative AKI (48.1% vs 16.0%, *p* < 0.001), postoperative LVEDD (48.78 ± 6.194 vs 47.25 ± 7.62 mm, *p* = 0.001), and red blood cell transfusion volume (3.04 ± 2.63 vs 2.45 ± 3.58 units, *p* = 0.006) significantly increased in the aortic occlusion length ≥90 minutes group (Table 1).

### 3.3 Analysis of Risk Factors of In-Hospital Mortality 

By univariable analysis, aortic occlusion length ≥90 minutes (odds ratio (OR): 2.870, 95% confidence interval (CI): 1.536–5.362, *p* = 0.001), preoperative neurological complications (OR: 3.917, 95% CI: 2.072–7.403, *p* < 0.001), vegetation length (OR: 1.126, 95% CI: 1.080–1.175, *p* < 0.001), and cardiac valve annulus destruction (OR: 6.125, 95% CI: 3.191–11.756, *p* < 0.001) were found to be related to in-hospital mortality. And by multivariable analyses, aortic occlusion length ≥90 minutes (OR: 2.916, 95% CI: 1.308–6.502, *p* = 0.009), preoperative neurological complications (OR: 3.676, 95% CI: 1.524–8.869, *p* = 0.004), vegetation length (OR: 1.170, 95% CI: 1.103–1.241, *p* < 0.001), and cardiac valve annulus destruction (OR: 2.770, 95% CI: 1.382–5.552, *p* = 0.004) were found to be related to in-hospital mortality (Table 2).

**Table 2.** **Analysis of risk factors for in-hospital mortality (n = 896)**.

Model		OR	95% CI	*p* value
Univariate analysis				
	Age	1.001	0.980–1.021	0.955
	Vegetation length	1.126	1.080–1.175	<0.001
	Pre-op LVEDD	0.989	0.959–1.020	0.476
	Aortic regurgitation	0.970	0.933–1.008	0.121
	Tricuspid regurgitation	1.034	0.969–1.104	0.315
	Aortic occlusion length ≥90 minutes	2.870	1.536–5.362	0.001
	Preoperative neurological complications	3.917	2.072–7.403	<0.001
	Cardiac valve annulus destruction	6.125	3.191–11.756	<0.001
Multivariate analysis				
	Vegetation length	1.170	1.103–1.241	<0.001
	Aortic occlusion length ≥90 minutes	2.916	1.308–6.502	0.009
	Preoperative neurological complications	3.676	1.524–8.869	0.004
	Cardiac valve annulus destruction	2.770	1.382–5.552	0.004

The survivors were further investigated. 814 survivors discharged from our medical center were successfully followed up and assigned to aortic occlusion length ≥90 minutes sub-group (n = 368) and aortic occlusion length <90 minutes sub-group (n = 446). Among the 896 IE patients who received surgical treatment, 48 died in the hospital (48/896, 5.36%), and the follow-up of 34 patients failed. The non-surgical mortality rate was 68.57% (768/1120) (Fig. 1).

### 3.4 Comparison Between Aortic Occlusion Length ≥ and <90 Minutes Sub-Groups

#### 3.4.1 Preoperative

Male gender (71.2% versus 62.3%, *p* = 0.008), age (41.05 ± 15.00 versus 36.17 ± 13.92 years, *p* < 0.001), duration between symptoms and operation (3.22 ± 3.25 versus 2.11 ± 1.77 months, *p* < 0.001), vegetation size (11.35 ± 6.67 versus 8.68 ± 6.47 mm, *p* < 0.001), preoperative LVEDD (62.43 ± 8.34 versus 59.73 ± 10.18 mm, *p* < 0.001), preoperative MR (10.64 ± 5.12 versus 4.42 ± 5.41 cm^2^, *p* < 0.001), vegetation size ≥10 mm (54.1% versus 30.9%, *p* < 0.001), aortic regurgitation preoperative ≥4 cm^2^ (54.6% versus 43.9%, *p* = 0.002), and time between symptoms and admission ≥2 months (83.4% versus 71.7%, *p* < 0.001) in aortic occlusion length ≥90 minutes sub-group were significantly higher than those in aortic occlusion length <90 minutes sub-group. Preoperative LVEF (59.57 ± 8.47 versus 61.74 ± 7.79%, *p* < 0.001) in the aortic occlusion length ≥90 minutes sub-group was significantly less than that in the aortic occlusion length <90 minutes sub-group (Table 3).

**Table 3.** **Preoperative data, surgical, and follow-up results**.

Variable		Aortic occlusion length ≥90 minutes sub-group (n = 368)	Aortic occlusion length <90 minutes sub-group (n = 446)	*p* value
Preoperative				
	Male gender, n (%)	262 (71.2%)	278 (62.3%)	0.008
	Age, years	41.05 ± 15.00	36.17 ± 13.92	<0.001
	Weight, kg	55.43 ± 11.23	56.42 ± 12.86	0.247
	Duration between symptoms and operation, months	3.22 ± 3.25	2.11 ± 1.77	<0.001
	Vegetation size, mm	11.35 ± 6.67	8.68 ± 6.47	<0.001
	Preoperative LVEDD, mm	62.43 ± 8.34	59.73 ± 10.18	<0.001
	Preoperative LVEF, %	59.57 ± 8.47	61.74 ± 7.79	<0.001
	Preoperative aortic regurgitation, cm^2^	5.78 ± 5.02	5.57 ± 7.82	0.684
	Preoperative mitral regurgitation, cm^2^	10.64 ± 5.12	4.42 ± 5.41	<0.001
	Preoperative tricuspid regurgitation, cm^2^	4.95 ± 3.25	4.53 ± 5.82	0.226
	Serum creatinine before surgery, µmol/L	81.27 ± 24.40	73.29 ± 21.04	0.342
	Aortic regurgitation preoperative ≥4 cm^2^, n (%)	201 (54.6%)	196 (43.9%)	0.002
	Vegetation size ≥10 mm, n (%)	199 (54.1%)	138 (30.9%)	<0.001
	Time between symptoms and operation ≥1 month, n (%)	307 (83.4%)	320 (71.7%)	<0.001
Operative				
	CPB time, minutes	179.33 ± 30.69	103.31 ± 23.98	<0.001
	Mechanical ventilation time, hours	48.19 ± 51.22	32.27 ± 33.88	<0.001
	Length of ICU stay, days	5.28 ± 2.91	3.89 ± 2.24	<0.001
	Postoperative hospital stay, days	20.82 ± 8.30	17.79 ± 6.01	<0.001
	Creatinine in serum 24 h after surgery, µmol/L	91.06 ± 33.48	83.04 ± 39.65	0.002
	Creatinine in serum 48 h after surgery, µmol/L	109.23 ± 56.79	84.51 ± 49.31	<0.001
	Chest drainage, mL	632.29 ± 465.49	603.72 ± 274.46	0.300
	Postoperative LVEDD, mm	48.68 ± 6.09	46.57 ± 7.79	<0.001
	Postoperative LVEF, %	57.90 ± 7.41	58.93 ± 7.82	0.056
	Frozen plasma transfusion, mL	634.39 ± 535.29	592.80 ± 372.52	0.208
	Red blood cells transfusion, units	3.05 ± 2.42	1.84 ± 2.27	<0.001
	CPB time ≥120 minutes, n (%)	368 (100.0%)	91 (20.4%)	<0.001
	Mechanical ventilation time ≥72 hours, n (%)	92 (25.0%)	45 (10.1%)	<0.001
	Length of ICU stay ≥3 days, n (%)	271 (73.6%)	152 (34.1%)	<0.001
	Postoperative LVEDD ≥70 mm, n (%)	77 (20.9%)	60 (13.5%)	<0.001
	Red blood cells transfusion ≥2 units, n (%)	230 (62.5%)	75 (16.8%)	<0.001
Follow up				
	Length of follow-up, months	62.73 ± 56.85	74.71 ± 47.87	0.001
	All-cause mortality in follow-up, n (%)	112 (30.4%)	21 (4.7%)	<0.001

#### 3.4.2 Operative

CPB time (179.33 ± 30.69 versus 103.31 ± 23.98 minutes, *p* < 0.001), mechanical ventilation time (48.19 ± 51.22 versus 32.27 ± 33.88 hours, *p* < 0.001), length of ICU stay (5.28 ± 2.91 versus 3.89 ± 2.24 days, *p* < 0.001), postoperative hospital stay (20.82 ± 8.30 versus 17.79 ± 6.01 days, *p* < 0.001), serum creatinine 48 h after surgery (91.06 ± 33.48 versus 83.04 ± 39.65 µmol/L, *p* = 0.002), serum creatinine 72 h after surgery (109.23 ± 56.79 versus 84.51 ± 49.31 µmol/L, *p* = 0.002), postoperative LVEDD (48.68 ± 6.09 versus 46.57 ± 7.79 mm, *p* < 0.001), red blood cells transfusion (3.05 ± 2.42 versus 1.84 ± 2.27 units, *p* < 0.001), CPB time ≥120 minutes (100.0% versus 20.4%, *p* < 0.001), mechanical ventilation time ≥72 hours (25.0%versus 10.1%, *p* < 0.001), length of ICU stay ≥3 days (73.6% versus 34.1%, *p* < 0.001), postoperative LVEDD ≥70 mm (20.9% versus 13.5%, *p* < 0.001), and red blood cells transfusion ≥2 units (62.5% versus 16.8%, *p* < 0.001) in aortic occlusion length ≥90 minutes sub-group were significantly higher than those in aortic occlusion length <90 minutes sub-group (Table 3).

#### 3.4.3 Follow-Up

The average follow-up time was 75.14 ± 1.80 months (range, 1 to 204). 87 cases (87/814, 10.7%) died within 12 months of discharge due to IE recurrence and cerebral hemorrhage. The latest follow-up data show that 681 survivors belong to New York Heart Association (NYHA) functional class I (681/727, 93.7%), and 46 survivors belong to class II (46/727, 6.3%). Length of follow-up (62.73 ± 56.85 versus 74.71 ± 47.87 months, *p* < 0.001) in the aortic occlusion length ≥90 minutes sub-group was statistically significantly less than that in the aortic occlusion length <90 minutes sub-group. Compared with the aortic occlusion length <90 minutes sub-group, all-time mortality in follow-up (30.4% versus 4.7%, *p* < 0.001) significantly increased in the aortic occlusion length ≥90 minutes sub-group (Table 3).

Operation and causes of in-hospital mortality and complications in IE were placed in the **Supplementary Material** and Table 4.

**Table 4.** **Operation and causes of in-hospital mortality and complications in infectious endocarditis (IE) (n = 896)**.

Variable		Value	Mortality
Operation			
	In-hospital mortality		5.36% (48/896)
	AVR isolated, %	19.64% (176/896)	1.34% (12/896)
	MVR isolated, %	41.07% (368/896)	1.79% (16/896)
	Double valve operation, %	28.57% (256/896)	2.23% (20/896)
	Bentall + MVR, %	1.79% (16/896)	0
	Tricuspid annuloplasty isolated, %	8.92% (80/896)	0
	ECMO, %	0.33% (3/896)	
Causes of in-hospital mortality, %			
	Paravalvular leak + septicemia + AKI + hepatic failure + cardiogenic shock	3.57% (32/896)	
	Intracerebral hemorrhage	1.79% (16/896)	
Complications			
	Mechanical ventilation time >72 h, %	21.43% (192/896)	
	Respiratory failure, %	15.07% (135/896)	
	AKI, %	28.68% (257/896)	
	Liver failure, %	4.35% (39/896)	
	Ventricular fibrillation, %	3.68% (33/896)	

AKI, acute kidney injury; AVR, aortic valve replacement; MVR, mitral valve replacement; ECMO, extracorporeal membrane oxygenation.

### 3.5 Risk Factors for Aortic Occlusion Length ≥90 Minutes 

By univariable analysis, duration between symptoms and operation ≥1 month (OR: 1.982, 95% CI: 1.406–2.794, *p* < 0.001), vegetation diameter ≥10 mm (OR: 2.682, 95% CI: 1.972–3.502, *p* < 0.001), CPB length ≥180 minutes (OR: 2.208, 95% CI: 1.451–2.835, *p* < 0.001), preoperative LVEDD ≥52 mm (OR: 1.932, 95% CI: 1.409–2.648, *p* < 0.001), preoperative aortic regurgitation ≥4 cm^2^ (OR: 1.535, 95% CI: 1.163–2.206, *p* < 0.001), and preoperative MR ≥8 cm^2^ (OR: 5.044, 95% CI: 3.740–6.802, *p* < 0.001) were showed to be associated with aortic occlusion length ≥90 minutes (Table 5).

**Table 5.** **Factors associated with aortic occlusion length ≥90 minutes (n = 896)**.

Model		OR	95% CI	*p* value
Univariate analysis				
	Duration between symptoms and operation ≥1 month	1.982	1.406–2.794	<0.001
	Vegetation diameter ≥10 mm	2.682	1.972–3.502	<0.001
	CPB length ≥180 minutes	2.208	1.451–2.835	<0.001
	Preoperative LVEDD ≥52 mm	1.932	1.409–2.648	<0.001
	Preoperative aortic regurgitation ≥4 cm^2^	1.535	1.163–2.206	0.002
	Preoperative MR ≥8 cm^2^	5.044	3.740–6.802	<0.001
Multivariate analysis				
	Duration between symptoms and operation	2.071	1.454–2.951	<0.001
	Vegetation diameter ≥10 mm	2.692	2.012-3.602	<0.001
	CPB length ≥180 minutes	1.690	1.150–2.481	<0.001
	Preoperative LVEDD ≥52 mm	4.164	3.868–15.042	<0.001
	Preoperative aortic regurgitation ≥4 cm^2^	5.211	3.431–7.914	<0.001
	Preoperative MR ≥8 cm^2^	12.980	8.437–19.969	<0.001

By multivariable analyses, duration between symptoms and operation ≥1 month (OR: 2.071, 95% CI: 1.454–2.951, *p* < 0.001), vegetation diameter ≥10 mm (OR: 2.692, 95% CI: 1.454–2.951, *p* < 0.001), CPB length ≥180 minutes (OR: 1.690, 95% CI: 1.150–2.481, *p* < 0.001), preoperative LVEDD ≥52 mm (OR: 4.164, 95% CI: 3.868–15.042, *p* < 0.001), preoperative aortic regurgitation ≥4 cm^2^ (OR: 5.211, 95% CI: 3.431–7.914, *p* < 0.001), and preoperative MR ≥8 cm^2^ (OR: 12.980, 95% CI: 8.437–19.969, *p* < 0.001) were showed to be associated with aortic occlusion length ≥90 minutes (Table 5).

### 3.6 Implications of Aortic Occlusion Length ≥90 Minutes

Univariable analysis indicated that aortic occlusion length ≥90 minutes is statistically significantly associated with 1-year mortality after cardiac operation (OR: 1.932, 95% CI: 1.406–2.794, *p* < 0.001) and 5-year mortality after cardiac operation (OR: 1.932, 95% CI: 1.409–2.648, *p* < 0.001), respectively (Table 4). Multivariable analysis indicated that aortic occlusion length ≥90 minutes is statistically significantly associated with 1-year mortality after cardiac operation (OR: 1.723, 95% CI: 1.200–2.474, *p* = 0.003) and 5-year mortality after cardiac operation (OR: 2.054, 95% CI: 1.454–2.901, *p* < 0.001), respectively (Table 6).

**Table 6.** **Implication of aortic occlusion length ≥90 minutes in infectious endocarditis (IE) (n = 814)**.

Model		OR	95% CI	*p* value
Univariate analysis of risk factors of 1-year mortality after cardiac operation (n = 87)				
	Aortic occlusion length ≥90 minutes	1.932	1.406–2.794	<0.001
Multivariate analysis of risk factors of 1-year mortality after cardiac operation (n = 87)				
	Aortic occlusion length ≥90 minutes	1.723	1.200–2.474	0.003
Univariate analysis of risk factors of 5-year mortality after cardiac operation (n = 100)				
	Aortic occlusion length ≥90 minutes	1.932	1.409–2.648	<0.001
Multivariate analysis of risk factors of 5-year mortality after cardiac operation (n = 100)				
	Aortic occlusion length ≥90 minutes	2.054	1.454–2.901	<0.001

### 3.7 Cox Proportional Hazard Regression for All-Time Mortality

Univariate analysis of Cox proportional hazard regression for all-time mortality identified duration between symptoms and operation ≥1 month (HR: 13.41, 95% CI: 4.246–41.922, *p* < 0.001), vegetation size ≥10 mm (HR: 3.264, 95% CI: 2.223–4.794, *p* < 0.001), aortic occlusion length ≥90 minutes (HR: 7.309, 95% CI: 4.583–11.656, *p* < 0.001), and red blood cell transfusion volume ≥2 units (HR: 2.648, 95% CI: 1.869–3.752, *p* < 0.001) to be associated with all-time mortality (Table 5). Multivariate analysis of Cox proportional hazard regression for all-time mortality identified duration between symptoms and operation ≥1 month (HR: 11.060, 95% CI: 3.474–35.218, *p* < 0.001), vegetation size ≥10 mm (HR: 3.031, 95% CI: 2.049–4.483, *p* < 0.001), aortic occlusion length ≥90 minutes (HR: 7.626, 95% CI: 4.681–12.424, *p* < 0.001), and red blood cell transfusion volume ≥2 units (HR: 3.362, 95% CI: 1.615–3.454, *p* < 0.001) to be associated with all-time mortality (Table 7).

**Table 7.** **Cox proportional risk regression of all-cause mortality during follow-up (n = 814)**.

Model		HR	95% CI	*p* value
Univariate analysis				
	Duration between symptoms and operation ≥1 month	13.410	4.246–41.922	<0.001
	Vegetation size ≥10 mm	3.264	2.223–4.794	<0.001
	Aortic occlusion length ≥90 minutes	7.309	4.583–11.656	<0.001
	Red blood cell transfusion volume ≥2 units	2.648	1.869–3.752	<0.001
Multivariate analysis				
	Duration between symptoms and operation ≥1 month	11.060	3.474–35.218	<0.001
	Vegetation size ≥10 mm	3.031	2.049–4.483	<0.001
	Aortic occlusion length ≥90 minutes	7.626	4.681–12.424	<0.001
	Red blood cell transfusion volume ≥2 units	3.362	1.615–3.454	<0.001

### 3.8 Association Between CPB Time and Aortic Cross-Clamping Time ≥90 Minutes

**Fig. 2.**
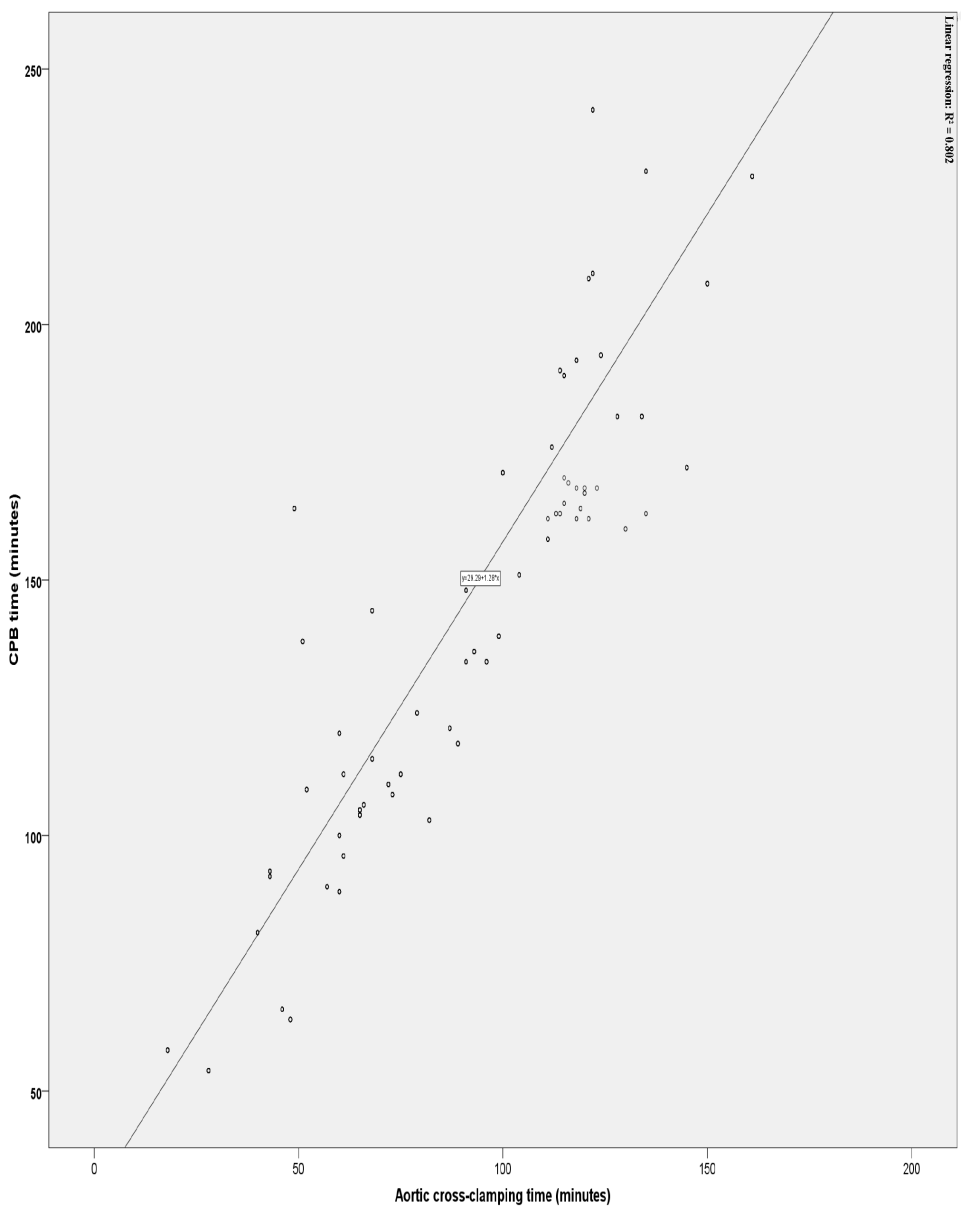
**Association between CPB time and aortic cross-clamping time ≥90 minutes**. A positive correlation between CPB time and aortic cross-clamping time exists.

Spearman correlation analysis showed a significant positive correlation between CPB time and aortic cross-clamping time (r = 0.895, *p* < 0.001) (Fig. 2).

### 3.9 The ROC Curve of Diagnostic CPB Time for Aortic Occlusion Length

**Fig. 3.**
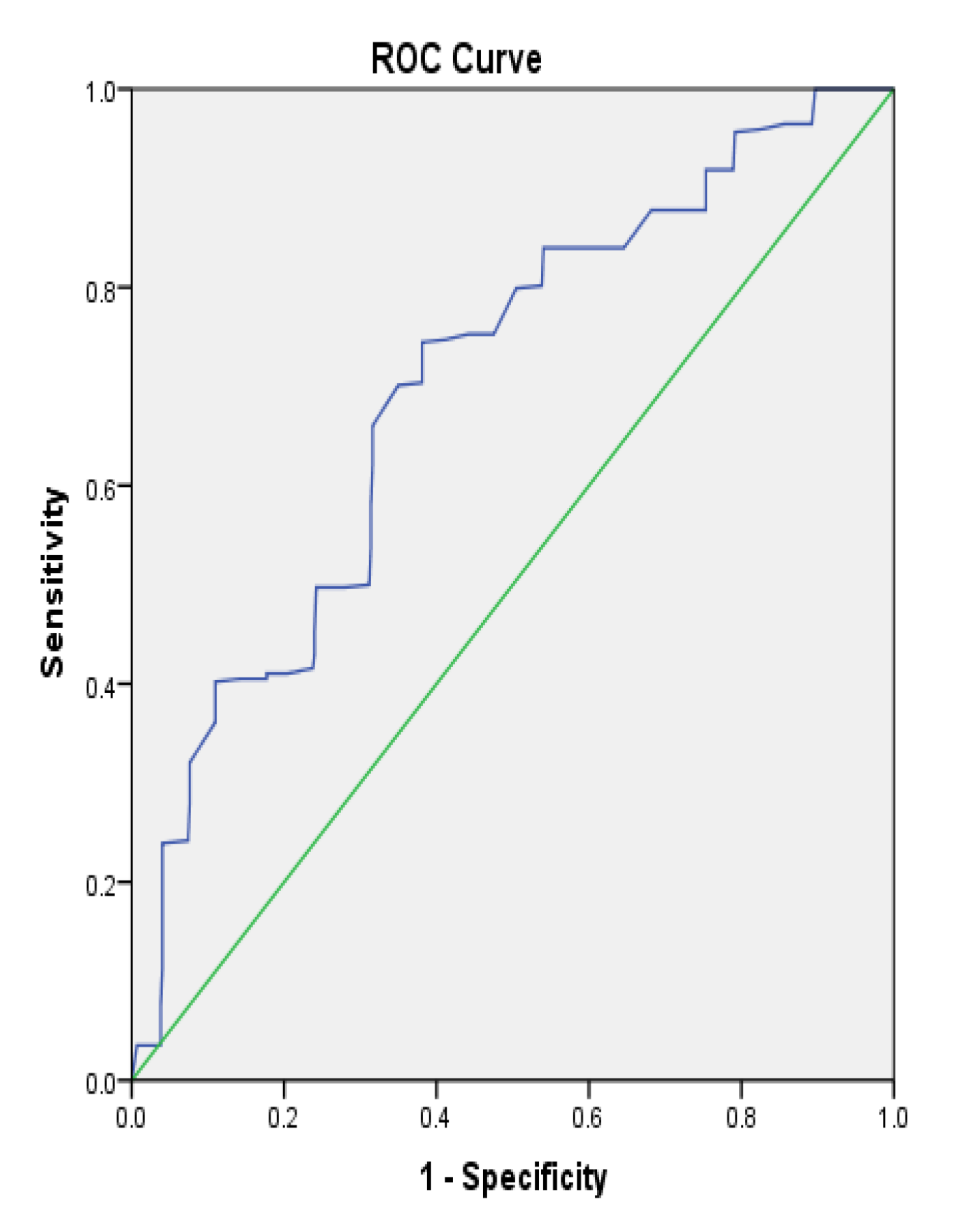
**The ROC curve of diagnostic CPB length for aortic occlusion length**. A value of CPB length >120.5 minutes was 74.5% sensitive and 61.9% specific for the diagnosis of aortic occlusion length ≥90 minutes, with an ROC curve of 0.702.

A value of CPB length >120.5 minutes was 74.5% sensitive and 61.9% specific for the diagnosis of aortic occlusion length ≥90 minutes, with an ROC curve 0.702 (95% CI: 0.666–0.737; *p* < 0.001). The Youden index was 0.364 (Fig. 3).

**Fig. 4.**
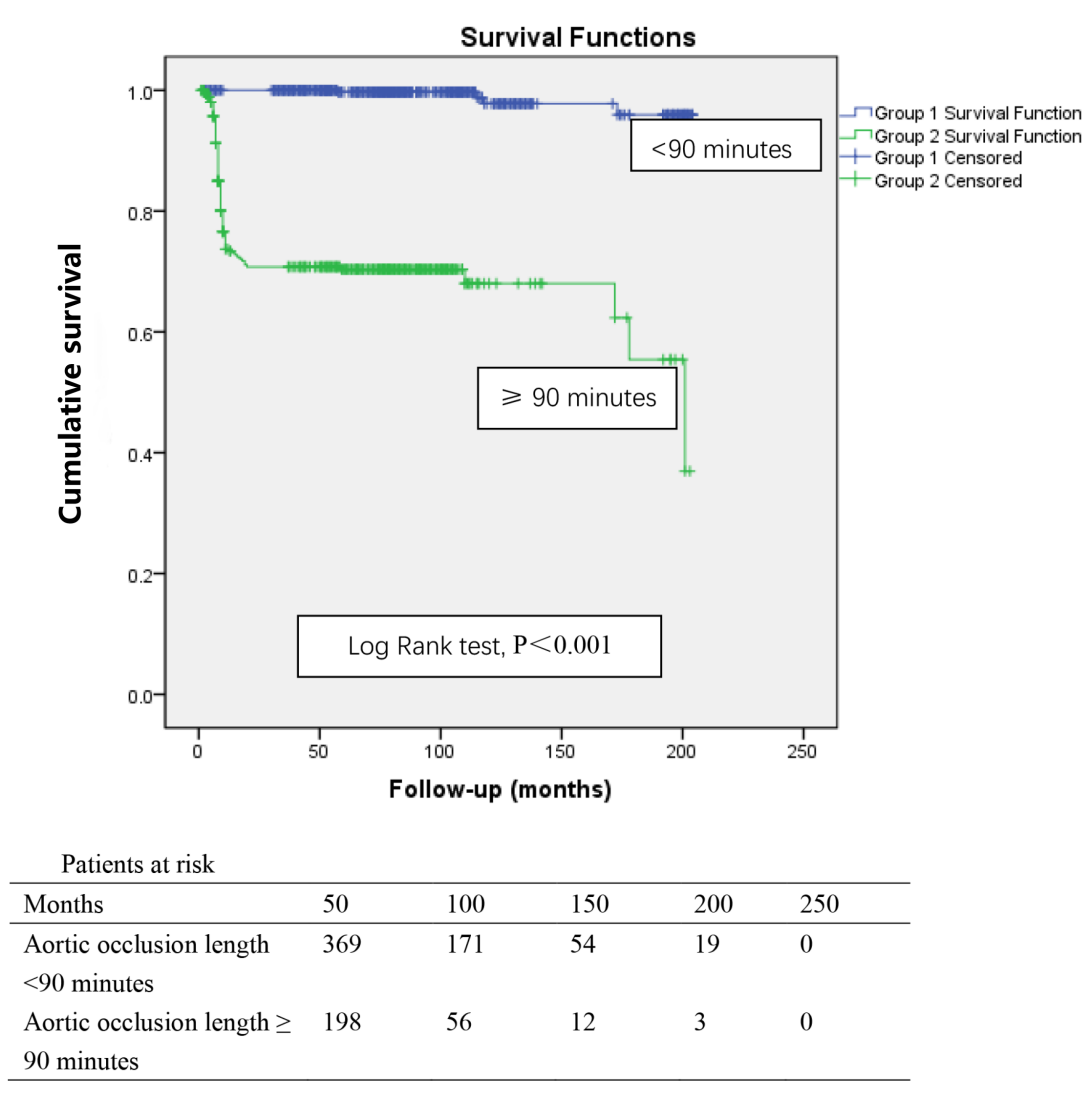
**Kaplan-Meier curve for survival**. The presence of aortic occlusion length ≥90 minutes is a significant risk factor for long-term survival. Blue line, aortic occlusion length <90 minutes; Green line, aortic occlusion length ≥90 minutes. Cum Survival, Cumulative Survival.

The presence of aortic occlusion length ≥90 minutes in IE is also a significant risk factor for long-term survival (Log-Rank test, *p* < 0.001) (Fig. 4).

## 4. Discussion

Myocardial protection is very important. It has been shown that the in-hospital mortality rate in the group of aortic occlusion length greater than 90 minutes was apparently greater than that in the group of aortic occlusion length ≤90 minutes. Research has shown that modern myocardial protection methods significantly reduce the expected mortality rate of long aortic occlusion. The mortality and incidence rate did not increase during long-term aortic occlusion with continuous retrograde warm cardioplegia and systemic normothermia [14,15,16,17,18]. It has been demonstrated that the duration of extracorporeal circulation is positively associated with interleukin-6 (IL-6) response, which is the cause of systemic inflammatory response associated with adverse results of heart operation. The cause of systemic inflammatory response is multifactorial, but there is evidence to suggest that contact between blood and artificial surfaces can result in the activation of platelets and cause changes in the shape of platelets. The alterations of shape activate a cascade reaction that produces inflammatory cytokines, complement, and coagulants. The use of continuous anterograde or retrograde infusion of warm, undiluted blood with or without added magnesium and potassium did not increase mortality in surgeries requiring aortic occlusion lengths exceeding two hours [19,20,21,22].

In the present research, there were 368 patients with aortic occlusion length ≥90 minutes (45.2%, 368/814). Aortic occlusion length ≥90 minutes was found to be associated with in-hospital mortality. Duration between symptoms and operation ≥1 month, vegetation diameter ≥10 mm, red blood cell transfusion volume ≥2 units, preoperative LVEDD ≥52 mm, preoperative aortic regurgitation ≥4 cm^2^, and preoperative MR ≥8 cm^2^ were shown to be associated with aortic occlusion length ≥90 minutes. Aortic occlusion length ≥90 minutes is apparently associated with 1- and 5-year mortality after cardiac operation. The presence of aortic occlusion length ≥90 minutes in IE is also apparently associated with long-term survival. Cox proportional hazard regression for all-time mortality identified duration between symptoms and operation ≥1 month, vegetation size ≥10 mm, aortic occlusion length ≥90 minutes, and red blood cell transfusion volume ≥2 units to be associated with all-time mortality. A value of CPB length >120.5 minutes was 74.5% sensitive and 61.9% specific for the diagnosis of aortic occlusion length ≥90 minutes. Vegetation diameter ≥10 mm, red blood cell transfusion volume ≥2 units, preoperative LVEDD ≥52 mm, preoperative aortic regurgitation ≥4 cm^2^, and preoperative MR ≥8 cm^2^ are parameters of disease severity.

In our study, the data in Table 1 and Table 3 clearly show that the aortic occlusion length ≥90 minutes group is a significantly sicker population at baseline: they are older, have larger vegetations, greater LVEDD, and more severe pre-operative regurgitation. By univariable analysis, age, vegetation size, pre-op LVEDD, aortic, and tricuspid regurgitation were not found to be associated with in-hospital mortality. By univariable and multivariable analysis, aortic occlusion length ≥90 minutes, preoperative neurological complications, and cardiac valve annulus destruction were found to be associated with in-hospital mortality (Table 2). Our study demonstrated that aortic occlusion length ≥90 minutes was an independent risk factor for in-hospital mortality. Early surgery and the establishment of a three-level prevention system are worth promoting to improve the long-term effectiveness of IE.

The condition of IE is complex and involves infections, cardiovascular system disorders, and even complications of other systems (e.g., acute renal failure, neurological complications, and splenic complications). The inflammatory burden of IE makes patients more susceptible to the inflammatory effects of CPB. With prolonged ischemia time, the levels of interleukin-6, interleukin-8, and interleukin-10 increased. Excessive length of aortic occlusion may increase inflammatory mediators, leading to an increased incidence of systemic inflammatory complications. Heparin-coated circuits and avoidance of cardiac incision aspiration have been applied to reduce inflammatory responses and mediators in inflammation. Correlation between prolonged aortic occlusion time and bypass duration with AKI has been reported [23,24,25]. AKI is multifactorial, including preoperative kidney function, hypoperfusion, embolism, and medication. Extracorporeal circulation time exceeding 90 minutes significantly increases renal dysfunction [26,27,28].

Extracorporeal circulation and aortic occlusion length are a few modifiable risk factors. The harmful effects of extracorporeal circulation and aortic occlusion length on renal function are multifactorial and well-known, and they are considered potential modifiable risk factors. CPB is associated with renal tubular injury, which increases the production of kidney-specific proteins such as neutrophil gelatinase-associated lipocalin, cysteine protease inhibitor C, and renal injury molecule 1. These proteins are discovered within 2–6 hours after surgery and are associated with the degree and duration of AKI as a biomarker. The longer the length of aortic occlusion, the lower the early survival rate after cardiac surgery [29,30,31,32,33].

The adverse effects of longer aortic occlusion length on early survival rates were associated with ischemia-induced myocardial injury, leading to a higher incidence of postoperative complications, including prolonged ventilation time, low cardiac output, higher transfusion requirements, and renal dysfunction. A longer length of aortic occlusion may have adverse biological effects on the human body and is independently associated with reduced late-stage survival [34,35,36]. Therefore, the length of aortic occlusion should be shortened as much as possible during cardiac surgery. Our investigation demonstrated the potential adverse effects of prolonged aortic occlusion time on late-stage survival. It may be wise to shorten the length of aortic occlusion as much as possible during cardiac surgery. We identified predictors (e.g., large vegetation, severe regurgitation) that can help surgeons anticipate complex cases requiring long aortic occlusion length, and the surgeons should take enough specific strategies (e.g., better myocardial protection, better surgical team preparation) to optimize all aspects of treatment in such scenarios [37,38,39,40]. Effective myocardial protection requires not only sufficient cardioplegia volume, but also uniform distribution. Antegrade cardioplegia is a physiological standard method; However, patients with significant coronary artery stenosis may have uneven distribution. In hypertrophic myocardium, increased vascular resistance may require increased infusion pressure and volume. Retrograde administration allows continuous operation, but may lead to uneven myocardial protection. In order to reduce the risk of inadequate protection, it is recommended to provide cardiac arrest fluid according to infusion time rather than volume. Although retrograde cardioplegia is helpful for continuous procedures, its effect on myocardial cooling may be low, which has aroused concern about the adequacy of protection [41,42,43]. Thermal imaging provides a practical and noninvasive method to evaluate myocardial temperature and detect insufficient cooling, and enhance myocardial protection strategies. Cardioplegic solution can be provided by anterograde perfusion, retrograde perfusion, or a combination of both. The choice of method varies from institution to institution. Anterograde perfusion follows a physiological route by inserting a needle into the aortic root or selectively into the left and right coronary artery orifices to deliver the solution. However, in patients with severe coronary heart disease, myocardial cooling may be inadequate or uneven. On the contrary, retrograde perfusion can protect the myocardium without interrupting the operation by delivering solution through a catheter inserted into the coronary sinus. Although this method is conducive to continuous surgery, it may not achieve uniform cooling of the entire myocardium. For complicated aortic valve replacement (AVR) and double valve replacement, we recommend using retrograde cardioplegia perfusion to shorten the time of aortic occlusion. In addition, histidine-tryptophan-ketoglutarate (HTK) solution or del Nido solution is recommended for good myocardial protection to obtain good short-term and long-term clinical effects. There should be a protocol for ensuring that a more senior or specialized lead surgeon scrubs in from the beginning for these high-risk cases [44,45,46].

## 5. Limitations 

The limitation of this study lies in its retrospective design, which may lead to selection bias due to the retrospective nature of the study and our hospital’s role as a tertiary referral center. Long-term recruitment of patients may have adverse effects on the outcome of cardiac surgery. Aortic occlusion length time is not randomly assigned; it is intrinsically linked to surgical complexity (e.g., double valve surgery, annular destruction, abscess). While we identify factors associated with aortic occlusion length ≥90 min, the multivariate models for mortality must adequately adjust for these underlying complexities, not just the factors leading to long aortic occlusion length. By univariable analysis, age, vegetation size, pre-op LVEDD, aortic, and tricuspid regurgitation were not found to be associated with in-hospital mortality. By univariable and multivariable analysis, aortic occlusion length ≥90 minutes, preoperative neurological complications, and cardiac valve annulus destruction were found to be associated with in-hospital mortality (Table 2). Our study demonstrated that aortic occlusion length ≥90 minutes was an independent risk factor for in-hospital mortality. Prospective randomized controlled trials are needed, and plans to reduce the incidence rate and mortality of nosocomial IE need to be developed.

## 6. Conclusions

In our investigation, aortic occlusion length ≥90 minutes in IE is a risk factor for long-term survival. It is wise to shorten the length of aortic occlusion as much as possible during cardiac surgery.

## Data Availability

The datasets used or analysed during the current study are available from the corresponding author on reasonable request.

## Abbreviations

IE, infectious endocarditis; NYHA, New York Heart Association; ICU, intensive care unit; Cm, Centimeter; Kg, kilogram; MR, mitral regurgitation; EF, ejection fraction; LVEDD, left ventricular end diastolic diameter; CPB, cardiopulmonary bypass; SD, standard deviation; RBC, red blood cells.
